# Detection of the G17V RHOA Mutation in Angioimmunoblastic T-Cell Lymphoma and Related Lymphomas Using Quantitative Allele-Specific PCR

**DOI:** 10.1371/journal.pone.0109714

**Published:** 2014-10-13

**Authors:** Rie Nakamoto-Matsubara, Mamiko Sakata-Yanagimoto, Terukazu Enami, Kenichi Yoshida, Shintaro Yanagimoto, Yusuke Shiozawa, Tohru Nanmoku, Kaishi Satomi, Hideharu Muto, Naoshi Obara, Takayasu Kato, Naoki Kurita, Yasuhisa Yokoyama, Koji Izutsu, Yasunori Ota, Masashi Sanada, Seiichi Shimizu, Takuya Komeno, Yuji Sato, Takayoshi Ito, Issay Kitabayashi, Kengo Takeuchi, Naoya Nakamura, Seishi Ogawa, Shigeru Chiba

**Affiliations:** 1 Department of Hematology, Graduate School of Comprehensive Human Sciences, University of Tsukuba, Tsukuba, Ibaraki, Japan; 2 Department of Hematology, Faculty of Medicine, University of Tsukuba, Tsukuba, Ibaraki, Japan; 3 Department of Hematology, University of Tsukuba Hospital, Tsukuba, Ibaraki, Japan; 4 Department of Pathology and Tumor Biology, Graduate School of Medicine, Kyoto University, Sakyo-ku, Kyoto, Japan; 5 Division for Health Service Promotion, The University of Tokyo, Bunkyo-ku, Tokyo, Japan; 6 Department of Clinical Laboratory, University of Tsukuba Hospital, Tsukuba, Ibaraki, Japan; 7 Department of Pathology, University of Tsukuba Hospital, Tsukuba, Ibaraki, Japan; 8 Life Science Center, Tsukuba Advanced Research Center, University of Tsukuba, Tsukuba, Ibaraki, Japan; 9 Department of Hematology, Toranomon Hospital, Minato-ku, Tokyo, Japan; 10 Okinaka Memorial Institute for Medical Research, Minato-ku, Tokyo, Japan; 11 Department of Pathology, Toranomon Hospital, Minato-ku, Tokyo, Japan; 12 Department of Hematology, Tsuchiura Kyodo General Hospital, Tsuchiura, Ibaraki, Japan; 13 Department of Hematology, Mito Medical Center, National Hospital Organization, Ibaraki-machi, Ibaraki, Japan; 14 Department of Hematology, Tsukuba Memorial Hospital, Tsukuba, Ibaraki, Japan; 15 Department of Hematology, JA Toride Medical Center, Toride, Ibaraki, Japan; 16 Division of Hematological Malignancy, National Cancer Center Research Institute, Chuo-ku, Tokyo, Japan; 17 Pathology Project for Molecular Targets, The Cancer Institute, Japanese Foundation for Cancer Research, Koto-ku, Tokyo, Japan; 18 Department of Pathology, Tokai University School of Medicine, Isehara, Kanagawa, Japan; University of North Carolina at Chapel Hill, United States of America

## Abstract

Angioimmunoblastic T-cell lymphoma (AITL) and peripheral T-cell lymphoma, not otherwise specified (PTCL-NOS) are subtypes of T-cell lymphoma. Due to low tumor cell content and substantial reactive cell infiltration, these lymphomas are sometimes mistaken for other types of lymphomas or even non-neoplastic diseases. In addition, a significant proportion of PTCL-NOS cases reportedly exhibit features of AITL (AITL-like PTCL-NOS). Thus disagreement is common in distinguishing between AITL and PTCL-NOS. Using whole-exome and subsequent targeted sequencing, we recently identified G17V *RHOA* mutations in 60–70% of AITL and AITL-like PTCL-NOS cases but not in other hematologic cancers, including other T-cell malignancies. Here, we establish a sensitive detection method for the G17V *RHOA* mutation using a quantitative allele-specific polymerase chain reaction (qAS-PCR) assay. Mutated allele frequencies deduced from this approach were highly correlated with those determined by deep sequencing. This method could serve as a novel diagnostic tool for 60–70% of AITL and AITL-like PTCL-NOS.

## Introduction

Based on the classification proposed by the World Health Organization (WHO), Angioimmunoblastic T-cell lymphoma (AITL) is a distinct subtype of T-cell lymphoma that accounts for 20% of peripheral T-cell lymphoma cases [1]. AITL is characterized by generalized lymphadenopathy, hyperglobulinemia, and autoimmune-like manifestations [1], [2]. Pathologic examination of AITL tumors reveals polymorphous infiltration of reactive cells, including endothelial venules and follicular dendritic cells [3], [4]. Based on gene expression profiling and immunohistochemical staining, the normal counterparts of AITL tumor cells are proposed to be follicular helper T cells (TFHs) [5]. Peripheral T-cell lymphoma, not otherwise specified (PTCL-NOS) is a more heterogenous type of lymphoma, one that shows variation even in CD4 and CD8 expression. Some PTCL-NOS cases share features of AITL, such as immunohistochemical staining patterns resembling those seen in AITL (AITL-like PTCL-NOS) [6].

Expertise is required to diagnose AITL and PTCL-NOS because generally low tumor cell content obscures the neoplastic nature of some cases, and large reactive B-cells are often confused with tumor cells [7]. Clonal rearrangement of the T-cell receptor gene is undetectable in 10–25% of AITL cases due to low tumor cell frequency [1]. In addition, clonal growth of Epstein-Bar virus-infected B-cells is not uncommon in these kinds of cancers, causing detection of clonal immunoglobulin gene rearrangement in 20% of these case. [1].

Mutations in *TET2, IDH2*, and *DNMT3A* are frequently seen in AITL and AITL-like PTCL-NOS [8], [9], although these mutations are also common to various myeloid malignancies [10], [11]. We and others reported a large cohort of AITL and PTCL-NOS patients revealing that the G17V *RHOA* mutation was highly specific to AITL and AITL-like PTCL-NOS and very frequent (seen in 60–70% of cases) in these T-cell lymphomas [12], [13]. This observation suggests that detection of the G17V *RHOA* mutation could serve as a new diagnostic tool to discriminate these lymphomas from other diseases. One difficulty, however, is that *RHOA* mutation allele frequencies in these lymphomas are generally as low as <0.2 or often <0.1, reflecting low tumor cell content. Therefore, diagnosis of these conditions requires development of sensitive and cost-efficient methods that are as accurate as deep sequencing, which is expensive and not commonly used in most clinical testing facilities.

To meet this need, we developed a quantitative allele-specific polymerase chain reaction (qAS-PCR) method that sensitively detects the G17V *RHOA* mutation in a highly accurate manner. This assay should provide a realistic way to conduct laboratory testing to diagnose AITL and AITL-like PTCL-NOS.

## Materials and Methods

### Primer design

**Figure 1:**
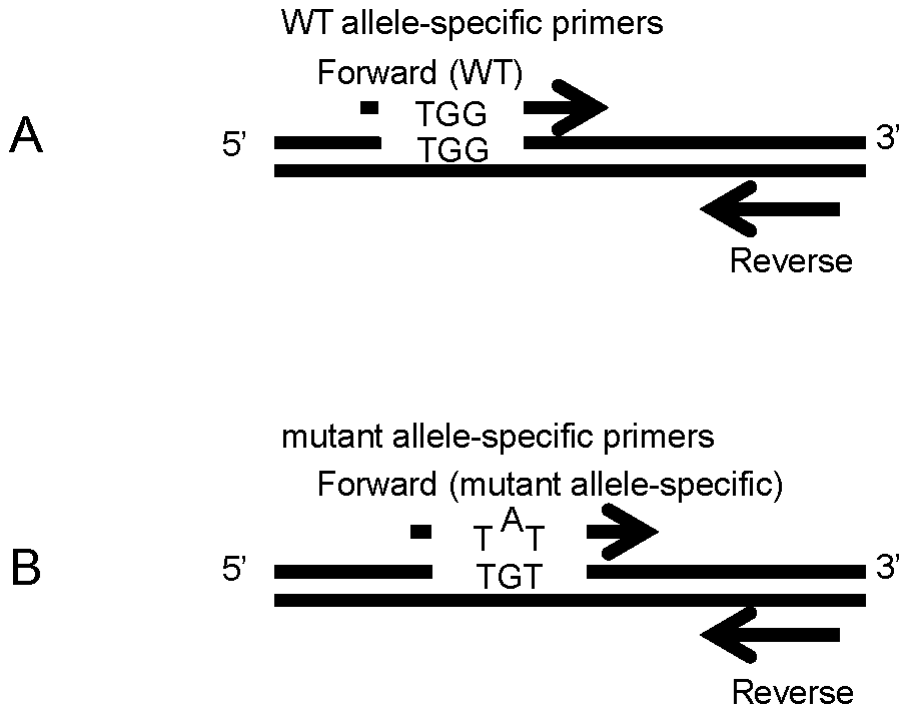
Design of primers used in the study. A WT allele-specific primer forward primer (Upper), a mutant allele-specific forward primer (Lower), and a common primer were designed. The 3′ end of the forward mutant primer was specific to the mutant site (G to T) and an internal mismatch at the second nucleotide from 3′ end (G to A) was introduced to improve specificity.

We designed two forward primers that discriminate wild-type (WT) from G17V *RHOA* for use with one common reverse primer. The mutant forward primer was designed using a previously described algorithm [14]. The 3′ end is specific to the mutant site and an internal mismatch at the second nucleotide from the 3′ end was introduced to improve specificity (Figure 1 and Table 1). We performed local alignment analysis using the BLAST program (http://www.ncbi.nlm.nih.gov/tools/primer-blast/) to confirm primer specificity.

**Table 1:** Sequence of allele-specific primers used for this study.

Primer	Sequence
Forward (WT* ^1^)	ATTGTTGGTGATGGAGCCTGTGG
Forward (MUT* ^2^)	ATTGTTGGTGATGGAGCCTGTAT
Reverse (common)	ACACCTCTGGGAACTGGTCCT

*^1^ WT, wild-type; *^2^ MUT, mutant.

### Preparation of plasmids containing WT and mutant cDNA and standard curve generation

WT or G17V mutant *RHOA* cDNA was subcloned into pBluescript (pBS/wtRHOA or pBS/mutRHOA, respectively; Agilent Technologies, Santa Clara, CA). qPCR reactions were performed in a final volume of 20 µl using 10 nM primers and the SYBR-Green mix (Roche Applied Science, Mannheim, Germany), and amplicons were subjected to either the ABI7500 or 7900 Fast Sequence Detection Systems (Life Technologies, Carlsbad, CA). Use of either the WT or mutant forward primer plus the common primer generated a 73-bp PCR product. The following PCR conditions were used: 10 min at 95°C, followed by 40 cycles of 15 sec at 95°C and 60 sec at 60°C.

Standard curves of amplicon levels were created by qPCR using serially-diluted pBS/wtRHOA or pBS/mutRHOA with WT or mutant primers, respectively.

### Preparation of template plasmid DNA mixtures

pBS/mutRHOA was mixed with pBS/wtRHOA in 100, 10, 1.0, 0.1, 0.01 and 0% ratios. Overall DNA concentration was adjusted to 1.0 ng/well of a plate. All mixtures were then serially-diluted 1∶10 for 4 cycles. qPCR was performed with these templates plus primers using conditions described above.

### Patients and samples

Tumor samples were collected from 53 patients with AITL, 55 with PTCL-NOS, 19 with B-cell malignancies, 129 with myeloid malignancies, and 5 with another T-cell lymphoma (for a total of 261), according to WHO classification. Twenty-seven non-tumor samples, including bone marrow mononuclear cells and buccal cells from lymphoma patients, were also analyzed as controls. The Ethics Committee University of Tsukuba Hospital approved the protocol and consent procedure, according to which written informed consent was provided by the participants. Genomic DNA was extracted from 13 formalin-fixed/paraffin-embedded (FFPE), 47 periodate/lysine/paraformaldehyde (PLP)-fixed, and 228 fresh frozen specimens, using an FFPE tissue kit (QIAGEN, Hilden, Germany) for FFPE and PLP samples and a Puregene DNA blood kit (QIAGEN) for fresh frozen specimens, according to manufacturer’s instructions.

One hundred and one DNA samples were original, while 187 were whole genome-amplified by either GenomiPhi (GE, Fairfield, CT) or a RepliG mini kit (Qiagen) (Table 2). For DNA extracted from FFPE samples, we also prepared PCR amplicon with AmpliTaq Gold 360 (Life technologies) in a final volume of 20 µl with 20 ng genomic DNA, 5 nM primers (Table 3), 5 µl of AmpliTaq gold master mix, and 0.3 µl of 360 GC Enhancer. For this amplicon preparation, the following PCR conditions were used: one cycle of 15 min at 95°C, 4 min at 60°C, and 1 min at 72°C, next 35 cycles of 1 min at 95°C, 1 min at 60°C, and 1 min at 72°C, and finally 10 min at 72°C and kept at 4°C. Amplicons were purified using PCR purification kit (QIAGEN).

**Table 2:** Analysis of genomic DNA samples.

Disease	Frozen amp* ^1^	Frozen not-amp* ^2^	PLP not-amp	FFPE not-amp	Total
AITL	14	10	19	10	53
PTCL-NOS	16	8	28	3	55
B-cell lymphoma	1	18			19
Myeloid malignancies	129				129
Other T-cell lymphomas		5			5
Control samples	27				27
Total	187	41	47	13	288

*^1^amp, amplified; *^2^not-amp, not-amplified.

**Table 3:** Primer sequences for making PCR amplicons of FFPE samples.

Primer	Sequence
Forward	GCCCCATGGTTACCAAAGCA
Reverse	GCTTTCCATCCACCTCGATA

Each DNA sample was quantified using the Qubit dsDNA HS Assay kit and a Qubit fluorometer (Life Technologies, Carlsbad, CA). Extracted DNA samples were stored at −20°C until use.

For 108 of the total 288 genomic DNA samples, data sets for mutant allele frequencies obtained by deep sequencing using the MiSeq System (Illumina, San Diego, CA), which were used in our previous report [12], were reanalyzed.

### qPCR of patient samples

qPCR reactions using duplicate patient samples were performed in a final volume of 20 µl with 50 ng of original or whole genome-amplified genomic DNA or 1.0×10^−2^ ng PCR-amplified DNA as a template, 10 nM primers, and the SYBR-Green mix (Roche, Basel, Switzerland) in conditions similar to those used for plasmid templates described above.

Levels of amplicons generated using either the WT or mutant primer, calculated with reference to respective standard curves, were designated [wt] and [mut], respectively.

### Statistical analysis

Statistical analysis was conducted using SPSS software (Japan International Business.

Machines Corporation, Tokyo). A P-value <0.05 was considered statistically significant.

## Results

### Primer specificity

**Figure 2:**
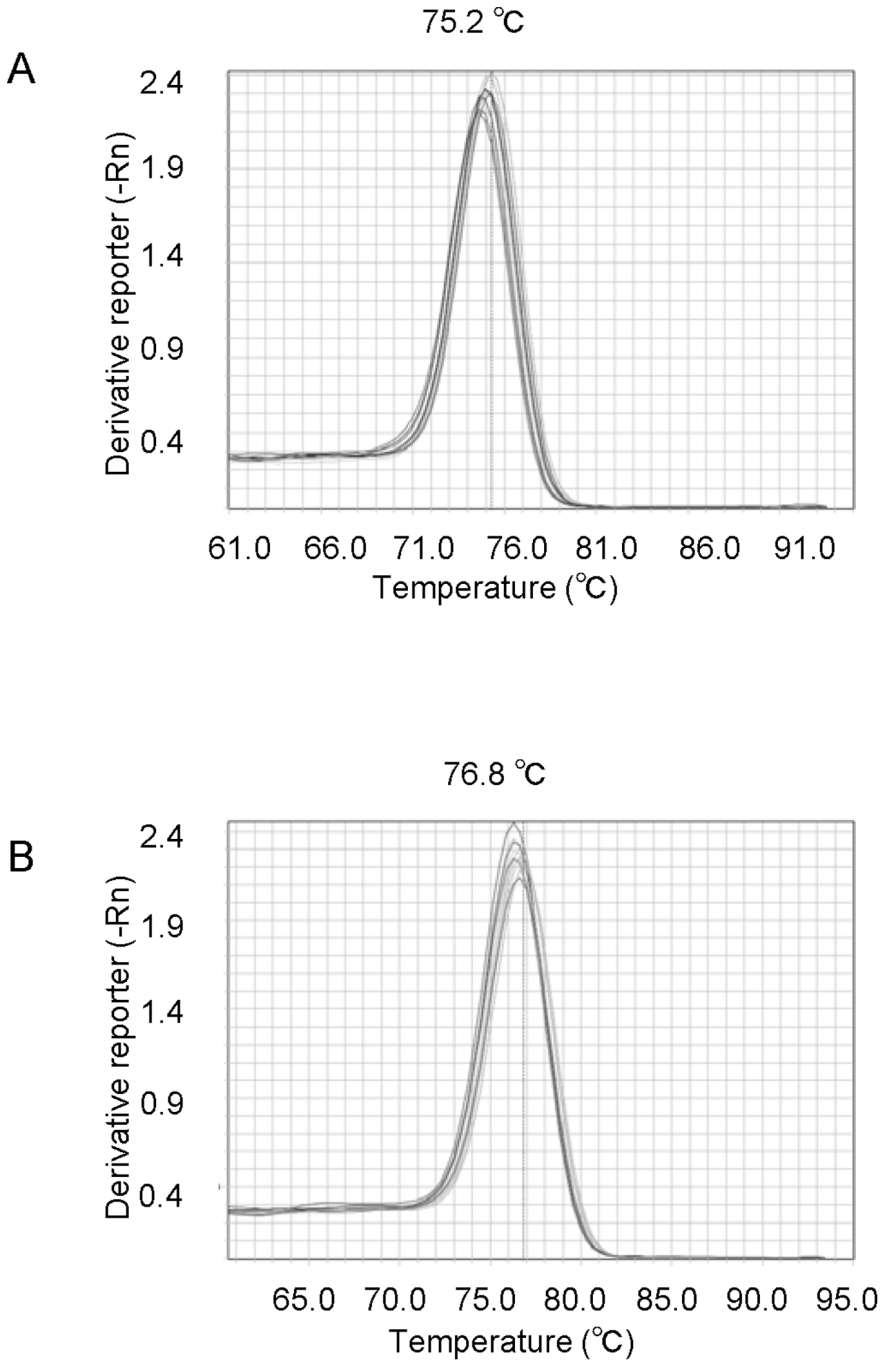
Melting curve analysis. A. Melting curve constructed using WT allele-specific primers. B. Melting curve constructed using mutant allele-specific primer set.

Melting curve analysis revealed that amplicons generated using either WT or mutant primers melted at 76.8°C or 75.3°C, respectively. Non-specific amplicons were not observed in either pBS/wtRHOA/WT primer or pBS/mutRHOA/mutant primer combinations (Figures 2A and 2B).

### Linearity of amplicon generation

**Figure 3:**
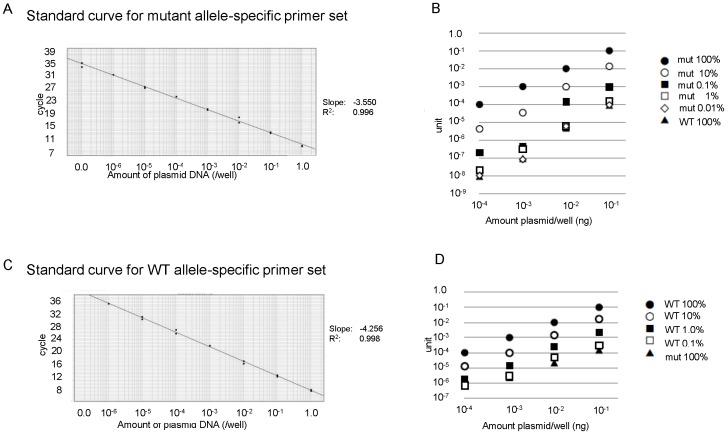
Standard curve showing linearity of quantitative allele-specific PCR. A standard curve was generated by serial dilution of WT or G17V cDNA that had been subcloned into pBluescript. A. Serial dilution of pBS/mutRHOA. Black dots correspond to 1.0×10^−9^∼1.0 unit of mutant plasmid (duplicate samples). The titration slope is −3.550 and R^2^ is 0.996. B. pBS/mutRHOA was mixed with pBS/wtRHOA at 100%, 10%, 1.0%, 0.1%, 0.01% and 0%. Mix concentrations were adjusted to 1.0 ng/well and diluted 1∶10 4 times for quantitative PCR analysis with allele-specific mutant primers. Horizonal axis indicates the amount of DNA per well. Vertical axis indicates unit for each sample. Black dot, MUT 100%; open dot, MUT 10%; square, MUT 1%; open square, MUT 0.1%; diamond, MUT 0.01%; triangle, MUT 0% (WT 100%) C. Serial dilution of pBS/wtRHOA. Black dots correspond to 1.0×10^−6^∼1.0 unit of WT cDNA (duplicate samples). The titration slope is −4.256, and R^2^ is 0.998. D. pBS/wtRHOA was mixed with pBS/mutRHOA at 100%, 10%, 1.0%, 0.1%, 0.1% and 0%. Mix concentrations were adjusted to 1.0 ng/well and diluted 1∶10 4 times for quantitative PCR analysis with WT allele-specific primers. Black dot, WT 100%; open dot, WT 10%; square, WT 1%; open square, WT 0.1%; triangle, WT 0% (MUT 100%).

We then varied either the ratio of pBS/mutRHOA to pBS/wtRHOA or the concentration of total input DNA, and measured the amounts of PCR product generated using the mutant primer. Because we observed a nearly linear relationship between the amounts of generated amplicon and input DNA in the range of 10^4^ (1–0.0001 ng DNA/well) at each ratio of pBS/mutRHOA to pBS/wtRHOA (Figure 3A), we defined the amount of amplicon derived from 100% pBS/mutRHOA template at 0.1 ng/well as 0.1 unit, and tested whether linearity was maintained with varying ratios of pBS/mutRHOA to pBS/wtRHOA. The template samples of 0.1 ng/well containing 10, 1, 0.1, and 0.01% pBS/mutRHOA were measured as 1.0×10^−2^ unit (C.I. (confidence interval), 0.8–1.3×10^−2^; S.F. (scaling factor), 0.95–1.06), 1.2×10^−3 ^unit (C.I., 0.8–1.6×10^−3^; S.F., 0.96–1.07), 2.2×10^−4^ unit (C.I., 1.5–3.0×10^−4^; S.F., 1.05–1.14), and 1.0×10^−5^ unit (C.I., 0.4–1.6×10^−5^; S.F., 0.92–1.04), indicative of linearity in the range of 10^4^ (100–0.01%). Taken together, linearity was maintained in the range of 10^9^ (Figures 3A and 3B).

Similarly, when we assessed the WT primer using various ratios of pBS/wtRHOA to pBS/mutRHOA and concentrations of input DNA, linearity between the amounts of amplicon and template were maintained between 100–0.1% (a range of 10^3^) and 1–0.001 ng DNA/well (a range of 10^3^). This analysis indicated a total dynamic range of 10^6^ (Figures 3C and 3D).

### qAS-PCR of T-cell lymphoma samples

**Figure 4:**
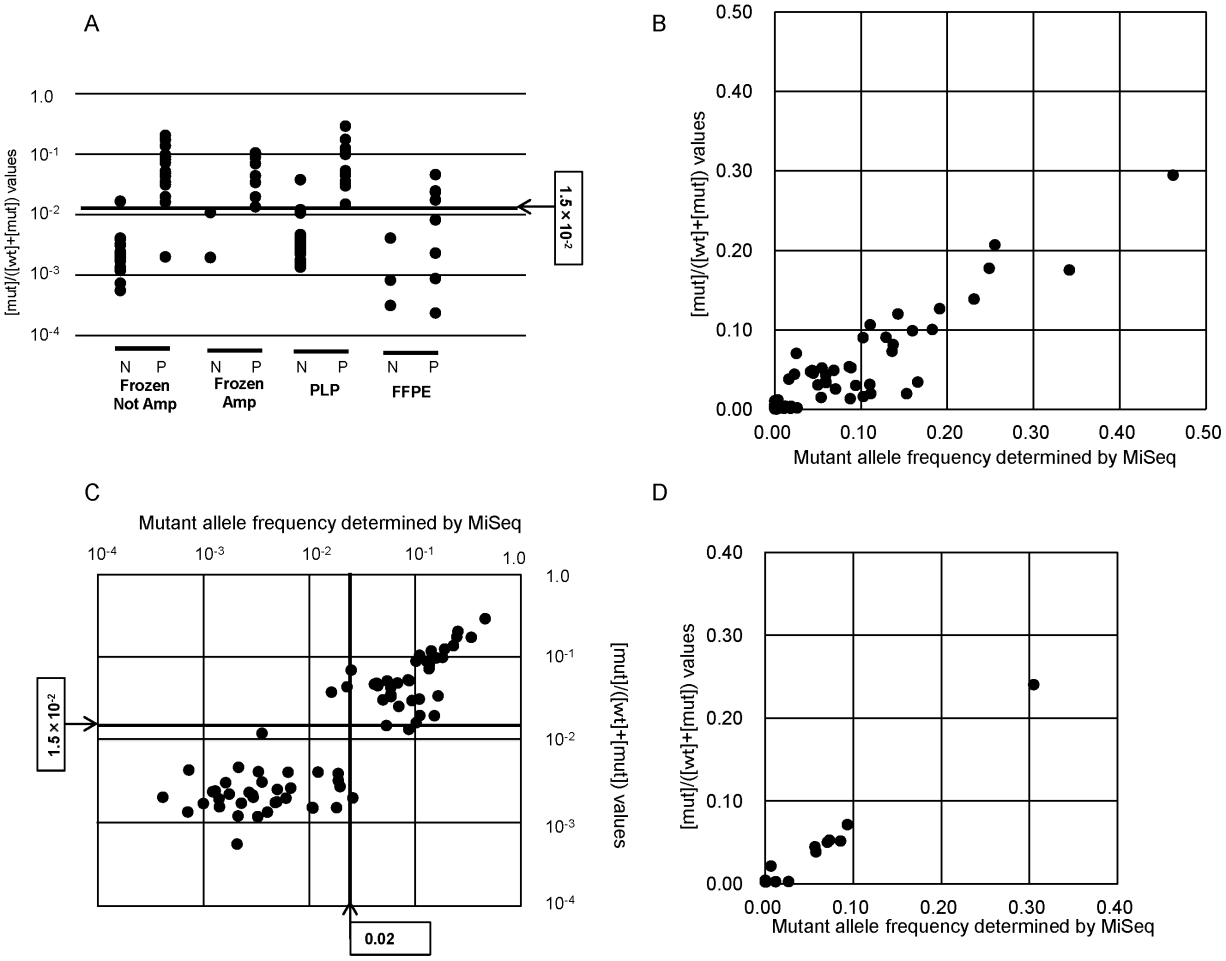
qAS-PCR of AITL and PTCL-NOS samples. A, Shown are [mut]/([wt]+[mut]) values for each sample. N, mutation negative determined by MiSeq; P, mutation positive determined by MiSeq; Amp, amplified; PLP, periodate/lysine/paraformaldehyde-fixed; FFPE, formalin-fixed/paraffin-embedded. B, Comparison of [mut]/([wt]+[mut]) values by qAS-PCR and mutant allele frequencies as determined by MiSeq for 95 original or whole genome-amplified DNA samples, including 43 AITL and 52 PTCL-NOS. Cut-off values were determined as 1.5×10^−2^ for [mut]/([wt]+[mut]) by qAS-PCR and as 0.02 for mutant allele frequencies as determined by MiSeq. C, Comparison of [mut]/([wt]+[mut]) values by qAS-PCR and mutant allele frequencies as determined by MiSeq for 95 DNA samples in a log scales. D, Comparison of [mut]/([wt]+[mut]) values by qAS-PCR and mutant allele frequencies as determined by MiSeq for 13 FFPE PCR-amplicon samples.

qAS-PCR with 50 ng of genomic DNA was performed using 106 AITL and PTCL-NOS samples including 11 FFPE samples. The [wt] and [mut] values were distributed between 7.9×10^−5^ and 1.8×10^−1^ units, and 2.0×10^−7^ and 7.6×10^−2^ units, respectively. Nevertheless, it was not possible to use absolute values of [mut] for levels of G17V *RHOA* alleles, due to variation in DNA quality. Therefore, we undertook relative measures to assess G17V *RHOA* allele frequency. To do so, we calculated a [mut]/([wt]+[mut]) value and compared it with mutant variant allele frequencies determined by MiSeq. [mut]/([wt]+[mut]) values were distributed between 3.2×10^−4^ and 3.0×10^−1^. Among samples judged to harbor a G17V *RHOA* mutation by deep sequencing using the MiSeq System (cut-off level, 0.02), which was defined in previous paper [12], [mut]/([wt]+[mut]) values of DNA from MiSeq-positive FFPE samples were significantly lower than those from other MiSeq-positive samples (Miseq-positive FFPE vs MiSeq-positive other samples; 1.56×10^−2^ vs. 9.38×10^−2^, p<0.05, Student's t-test) (Figure 4A). Four out of all 8 MiSeq-positive FFPE samples were negative by qAS-PCR. Therefore, we excluded FFPE samples and analyzed data from 95 DNA samples that had been purified from PLP-fixed or frozen tissues.

When [mut]/([wt]+[mut]) values were compared with mutant variant allele frequencies determined by MiSeq, the rank correlation coefficient was 0.785 (Spearman’s correlation P<0.001) (Figure 4B and C). Among the 95 samples analyzed, 38 (29 AITL and 9 PTCL-NOS) were judged positive and 57 (14 AITL and 43 PTCL-NOS) were judged negative by MiSeq. By comparison, when the cut-off level for [mut]/([wt]+[mut]) values was set at 1.5×10^−2^, according to ROC curve (Supplemental Figure 1), 38 cases were judged positive for the G17V *RHOA* mutation, including 29 AITL and 9 PTCL-NOS. Overall, 91 of 95 specimens showed concordant results using both methods, while 4 cases showed discordant results (Figure 4B and C). If we assume that data generated by MiSeq is accurate, then the sensitivity and specificity of qAS-PCR were as high as 94.7% and 96.5%, respectively. Positive and negative concordance rates of the two methods were 94.7% and 96.5%, respectively (Table 4, Table S1 in File S1).

**Table 4:** Correlation between qAS-PCR and MiSeq.

Method	Standard	Samples			N* ^1^	RCC* ^2^	Sensitivity	Specificity	PPV* ^3^	NPV* ^4^
qAS-PCR	MiSeq	AITL and PTCL-NOS	non- FFPE	all	95	0.785	94.7	96.5	94.7	96.5
				original	66	0.735	100.0	95.5	91.7	100.0
				WGA* ^5^	29	0.822	87.5	100.0	100	86.7
			FFPE * ^6^		13	0.919	87.5	80.0	87.5	80.0

*^1^N, number; *^2^RCC, rank correlation coefficient; *^3^PPV, positive predictive value; *^4^NPV, negative predictive value, *^5^WGA, whole-genome amplification. *^6^FFPE, formalin-fixed/paraffin-embedded.

The four cases showing discordant results provided us with an insight into the comparison between MiSeq and aAS-PCR. Two samples were positive only based on MiSeq, and two were positive only by qAS-PCR. When we performed HISEQ2000 sequencing [12] for all these four samples, we observed ≧0.02 mutation allele frequencies in two samples. One had been deemed positive only by qAS-PCR and the other only by MiSeq. The other two samples showed <0.02 mutation allele frequencies by HISEQ2000. One of them was judged as negative only by qAS-PCR and the other only by MiSeq. Overall, accuracy with qAS-PCR and MiSeq was comparable.

The qAS-PCR method using 50 ng of whole-genome-amplified DNA did not provide a robust correlation with the Miseq data for FFPE samples. The main reason was likely to be fragmentation of genomic DNA. To overcome this limitation, DNA prepared from the 13 FFPE samples was pre-amplified by PCR prior to performing qAS-PCR. Sensitivity and specificity for FFPE samples using amplicon was 87.5% and 80.0%, respectively, based on the mutation allele frequencies determined by MiSeq. (Figure 4D, Table S2 in File S1). Therefore, even for FFPE samples, the qAS-PCR method could robustly estimate the G17V RHOA mutation allele frequencies.

### Effect of whole-genome amplification for qAS-PCR

When we divided the 95 samples into original DNA and whole-genome-amplified DNA cohort, sensitivity and specificity were 100% and 95.5% for original DNA cohort, and 87.5% and 100% for whole-genome-amplified DNA cohort, respectively (Supplemental Figure 2A-D, Table S3A and B in File S1).

In order to determine whether amplification influences the evaluation of mutation allele frequency by qAS-PCR, we compared the data for 15 pairs of original and whole-genome-amplified samples. Fourteen out of 15 pairs showed concordant results with each other (Table S3C and D in File S1, Figure S2E in File S1). One sample, which was judged positive by MiSeq, showed discordant results by qAS-PCR; positive for the original DNA and negative for the whole-genome-amplified DNA. As a summary, with some limitations, whole-genome-amplified DNA could provide robust results in most cases.

### qAS-PCR for myeloid, B-cell and other T-cell malignancies

**Figure 5:**
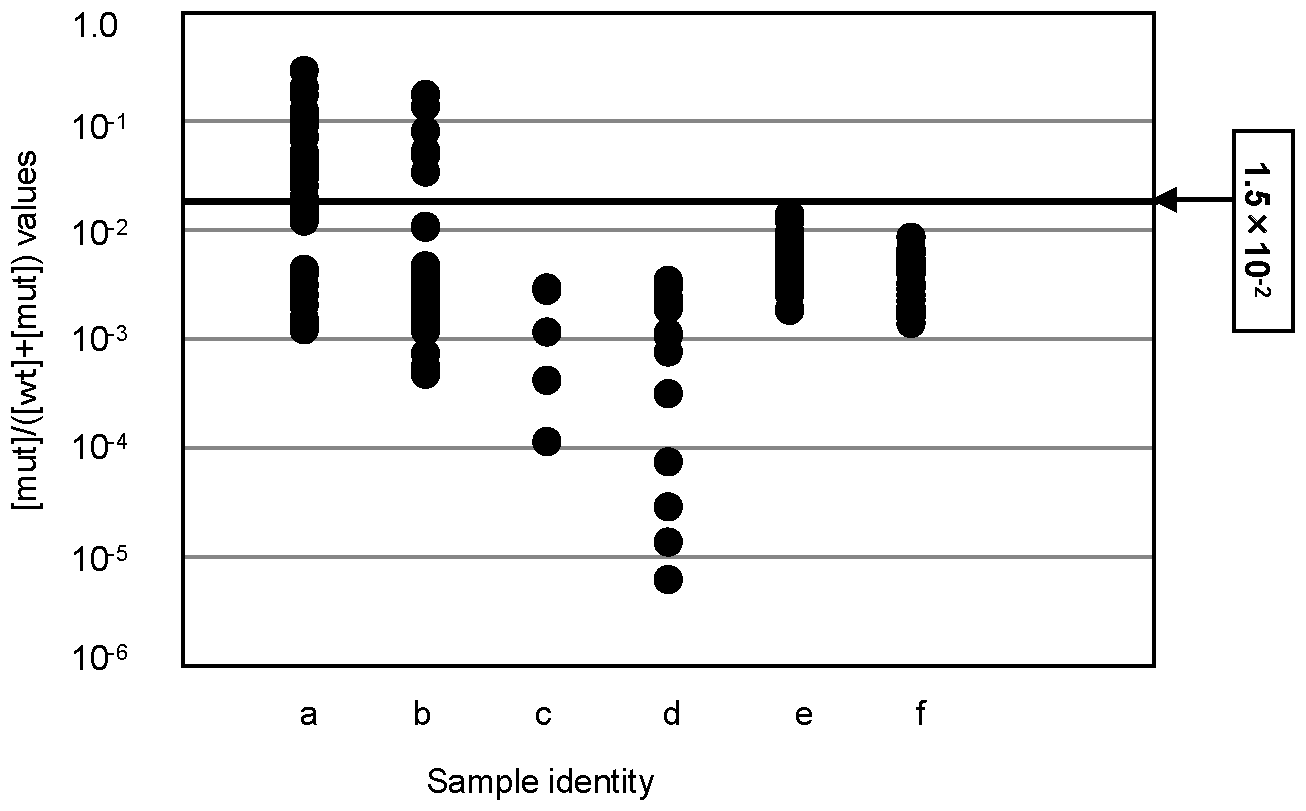
qAS-PCR for 275 tumor and control samples. qAS-PCR was performed for tumor samples, including 43 AITL (a), 52 PTCL-NOS (b), 5 T-cell lymphoma other than AITL and PTCL-NOS (c), 19 B-cell lymphomas (d), 129 myeloid malignancies (e) and 27 control samples (f).

We performed qAS-PCR for buccal cells and non-tumor samples including bone marrow cells without lymphoma infiltration obtained from lymphoma patients, and confirmed that the qAS-PCR values were below the cut-off level in all samples. Then, we applied qAS-PCR for 153 tumor samples other than AITL and PTCL-NOS, including 129 myeloid, 19 B-cell, and 5 T-cell malignancies. Sanger sequencing also showed no mutant signals for any of these samples. All qAS-PCR values calculated using these samples were below the cut-off level (Figure 5).

## Discussion

Our recent discovery of the highly frequent G17V *RHOA* mutation in AITL and AITL-like PTCL-NOS led us to develop a novel method to detect this mutation [12]. The results of qAS-PCR analysis described here are correlated well with those derived from deep sequencing (Table 4), while qAS-PCR is superior to deep sequencing in terms of the cost and convenience. There is a pressing clinical need for a well-validated *RHOA* testing method with optimal analytical performance using the least amount of difficult-to-obtain patient specimens. We show here that even DNA samples subjected to whole-genome amplification or low quality/concentration DNA extracted from FFPE samples can serve as reliable material for our qAS-PCR method, if appropriate PCR procedure and primers are used. Allele-specific PCR for G17V *RHOA* mutation was mentioned in other report [13], although sensitivity and specificity of the methods were not described.

In a previous study, we defined the cut-off level of mutant allele frequencies determined by MiSeq as 0.02 [12]. In this study, we defined the cut-off level as 1.5×10^−2^ for qAS-PCR, but it remains to be determined whether these cut-off levels are sufficient to detect AITL. Given our finding that the mutated *RHOA* allele frequencies distributed below 0.05 in many AITL samples [12], the tumor cell content might be very low and could be detected in some cases only when the cut-off levels of qAS-PCR and deep sequencing are lowered. If we set the cut-off value lower, the sensitivity should be improved with the increase of false-positive results, raising a dilemma common to other clinical testings.

Several hotspot mutations that reveal distinct hematologic malignancies have been identified in conditions other than T-cell lymphomas. For example, detection of the V617F *JAK2* mutation is a part of the diagnostic criteria for myeloproliferative neoplasms in the latest version of WHO classification [1], although consensus is not reached about the detection methods and cut-off levels. Methods have been developed to detect this mutation including allele-specific PCR and a PCR-restriction fragment length polymorphism (RFLP) approach utilizing mutation sequence specificity for a restriction enzyme[15]–[18]. More recently, a V600E *BRAF* mutation in hairy cell leukemia [19], an L265P *MYD* mutation in Waldenström macrogloblinemia [20], and several mutations in *STAT3* in large granular lymphocytic leukemia [21] have been identified as diagnostics of these tumor types. In the future, it is likely that molecular alterations, including the G17V *RHOA* mutation, will be increasingly incorporated into the diagnostic criteria for hematologic malignancies. In summary, our novel method to detect the G17V *RHOA* mutation could provide an important clinical tool to diagnose AITL and AITL-like PTCL-NOS and in the future serve as a means to classify AITL and PTCL-NOS.

## Supporting Information

File S1 **Figures S1–S2 and Tables S1–S4.** Figure S1. ROC curve for data of qAS-PCR and MiSeq. Horizontal axis shows 1-specificity and Vertical axis shows sensitivity of qAS-PCR method compared to the data of MiSeq. Figure S2. Effect of whole-genome amplification for qAS-PCR A, Comparison of [mut]/([wt]+[mut]) values by qAS-PCR and mutant allele frequencies as determined by MiSeq for 66 original samples (linear). B, Comparison of [mut]/([wt]+[mut]) values by qAS-PCR and mutant allele frequencies as determined by MiSeq for 66 original samples (log scale). C, Comparison of [mut]/([wt]+[mut]) values by qAS-PCR and mutant allele frequencies as determined by MiSeq for 29 whole-genome amplified samples (linear). D, Comparison of [mut]/([wt]+[mut]) values by qAS-PCR and mutant allele frequencies as determined by MiSeq for 29 whole-genome amplified samples (log scale). E, Comparison of [mut]/([wt]+[mut]) values by qAS-PCR for 15 pairs of original and whole-genome amplified samples in a log scale.
(PDF)
