# Supplementary material for: Detection of the G17V RHOA Mutation in Angioimmunoblastic T-Cell Lymphoma and Related Lymphomas Using Quantitative Allele-Specific PCR
Source: PLoS One. 2014 Oct 13;9(10):e109714. doi: 10.1371/journal.pone.0109714 (PMC4195681; doi:10.1371/journal.pone.0109714)

Supplemental Information

Detection of the G17V RHOA mutation in angioimmunoblastic T-cell lymphoma and related lymphomas using quantitative allele-specific PCR

Rie Nakamoto-Matsubara, Mamiko Sakata-Yanagimoto, Terukazu Enami, Kenichi Yoshida, Shintaro Yanagimoto, Yusuke Shiozawa, Tohru Nanmoku, Kaishi Satomi, Hideharu Muto, Naoshi Obara, Takayasu Kato, Naoki Kurita, Yasuhisa Yokoyama, Koji Izutsu, Yasunori Ota, Masashi Sanada, Seiichi Shimizu, Takuya Komeno, Yuji Sato, Takayoshi Ito, Issay Kitabayashi, Kengo Takeuchi, Naoya Nakamura, Seishi Ogawa and Shigeru Chiba

---

Section

Supplemental Tables

Supplemental Figures

Supplemental Tables

Supplemental Table 1

Comparison between [mut]/ ([wt]+[mut]) by qAS-PCR and allele frequency determined by MiSeq for original and whole-genome amplified samples.

	MiSeq+	MiSeq-	Total
qAS-PCR+	36	2	38
qAS-PCR-	2	55	57
total	38	57	95

Supplemental Table 2

Comparison between [mut]/ ([mut]+ [wt]) by PCR amplicon-based qAS-PCR and mutant allele frequency determined by MiSeq for 13 FFPE samples.

	MiSeq+	MiSeq-	total
qAS-PCR+	7	1	8
qAS-PCR-	1	4	5
total	8	5	13

Supplemental Table 3

A. Comparison between [mut]/ ([wt]+[mut]) by qAS-PCR and allele frequency determined by MiSeq for 66 original samples.

	MiSeq+	MiSeq-	total
qAS-PCR+	22	2	24
qAS-PCR-	0	42	42
total	22	44	66

B. Comparison between [mut]/ ([wt]+[mut]) by qAS-PCR and allele frequency determined by MiSeq for 29 whole-genome amplified samples.

	MiSeq+	MiSeq-	total
qAS-PCR+	14	0	14
qAS-PCR-	2	13	15
total	16	13	29

C. Comparison between [mut]/ ([wt]+[mut]) by qAS-PCR and mutant allele frequency determined by MiSeq for 13 original sample.

	MiSeq+	MiSeq-	Total
qAS-PCR+	6	0	6
qAS-PCR-	0	9	9
total	6	9	15

D. Comparison between [mut]/ ([wt]+[mut]) by qAS-PCR and mutant allele frequency determined by MiSeq for 13 whole-genome amplified sample.

	MiSeq+	MiSeq-	Total
qAS-PCR+	5	0	5
qAS-PCR-	1	9	10
total	6	9	15

Supplemental Figure 1

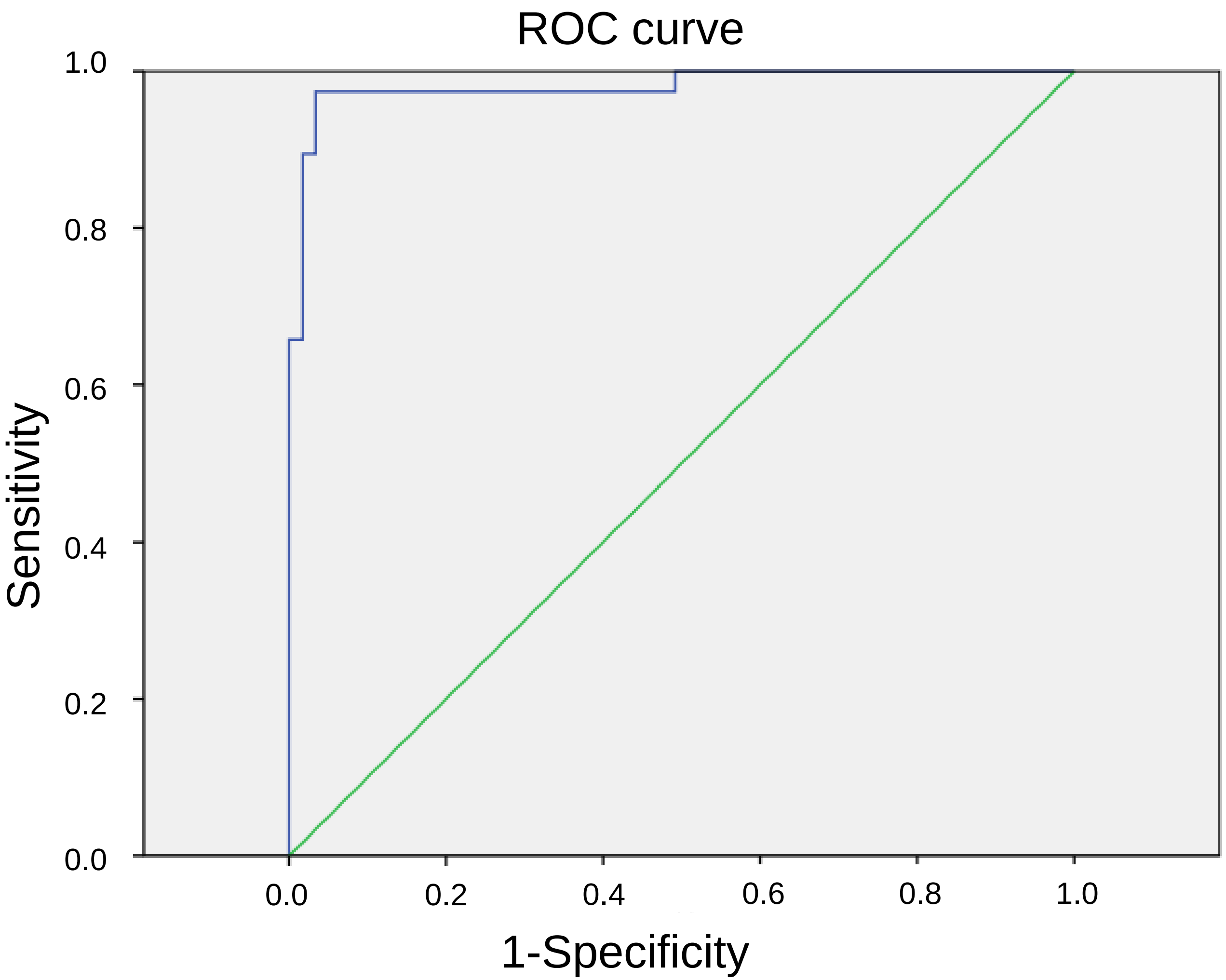

Supplemental Figure 2

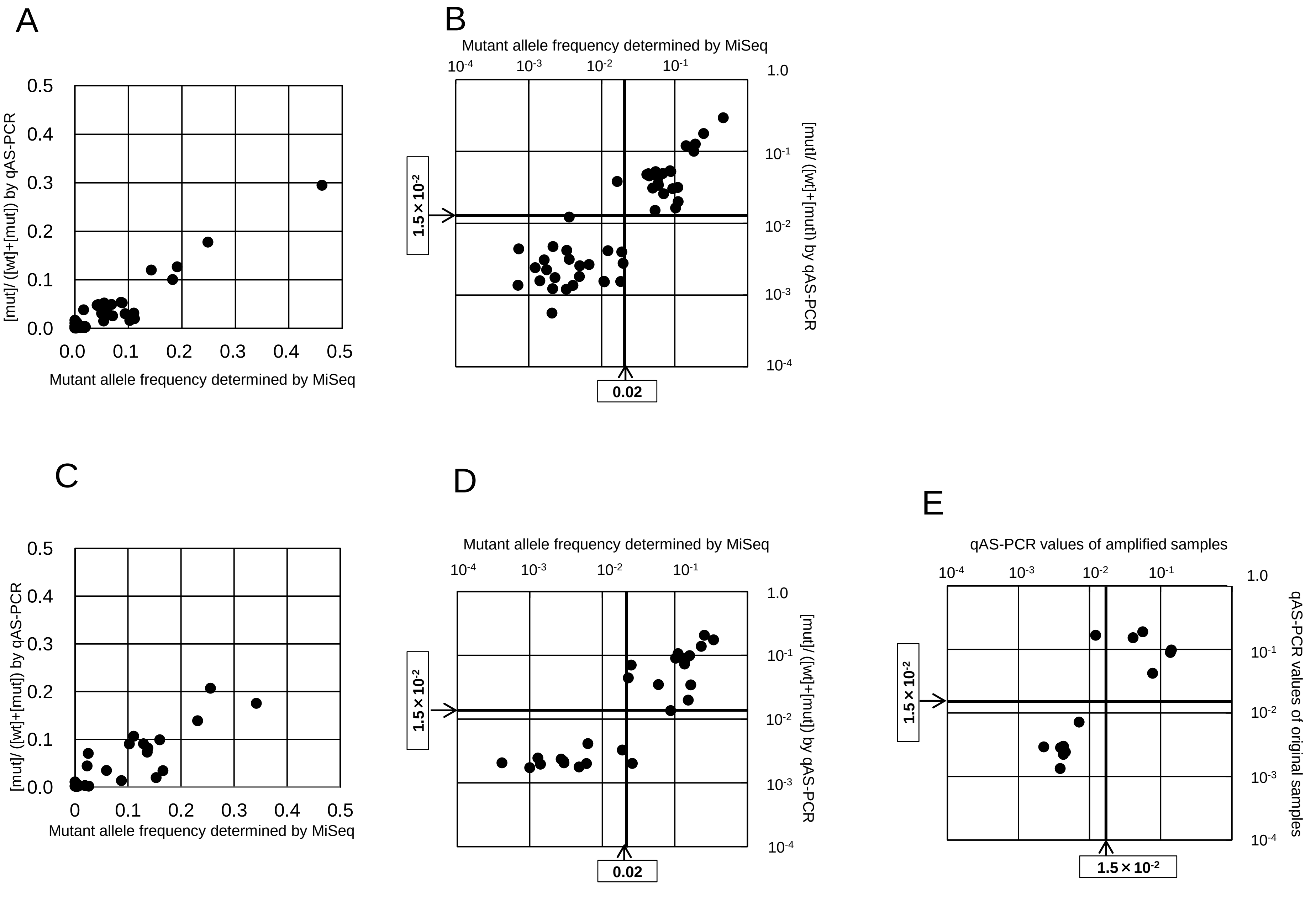

Supplement: File S1 — Figures S1–S2 and Tables S1–S4. Figure S1. ROC curve for data of qAS-PCR and MiSeq. Horizontal axis shows 1-specificity and Vertical axis shows sensitivity of qAS-PCR method compared to the data of MiSeq. Figure S2. Effect of whole-genome amplification for qAS-PCR A, Comparison of [mut]/([wt]+[mut]) values by qAS-PCR and mutant allele frequencies as determined by MiSeq for 66 original samples (linear). B, Comparison of [mut]/([wt]+[mut]) values by qAS-PCR and mutant allele frequencies as determined by MiSeq for 66 original samples (log scale). C, Comparison of [mut]/([wt]+[mut]) values by qAS-PCR and mutant allele frequencies as determined by MiSeq for 29 whole-genome amplified samples (linear). D, Comparison of [mut]/([wt]+[mut]) values by qAS-PCR and mutant allele frequencies as determined by MiSeq for 29 whole-genome amplified samples (log scale). E, Comparison of [mut]/([wt]+[mut]) values by qAS-PCR for 15 pairs of original and whole-genome amplified samples in a log scale. (PDF) [file pone.0109714.s001.pdf]
